# App-supported Intervention for Digital Aftercare to Bridge Long Waiting Periods between Inpatient and Outpatient Treatment of Depression

**DOI:** 10.1192/j.eurpsy.2026.11427

**Published:** 2026-07-31

**Authors:** K. Friedrich, J. Krieger, V. Rößner-Ruff, C. Penkov, M. Ziegenbein

**Affiliations:** Research & Development, Wahrendorff Klinikum, Sehnde, Germany

## Abstract

**Introduction:**

Given the elevated risk of relapse following the completion of acute inpatient depression treatment until remission, the guidelines advise the use of maintenance therapy or continuation over several months, with a focus on relapse prevention. Digital health technologies can facilitate support for patients at the vulnerable interface when changing from partial inpatient treatment to further outpatient treatment, therby contributing to a successful transition between healthcare sectors. Accordingly, an app-supported intervention was examined. The app was utilized as a communication platform for this purpose. A distinguishing aspect of this approach is the additional psychotherapeutic assistance provided to patients via the app by their therapists after they have been discharged from the day clinic. The application enables sharing individualized therapy content and facilitates communication via a chat function.

**Objectives:**

The objective of the present study is to investigate whether the app-intervention can provided continuity of care despite prolonged waiting periods when a transition is made from part-time inpatient to outpatient care.

**Methods:**

This is a longitudinal intervention study. Depressive symptoms (BDI-II) and quality of life (WHOQOL_BREF) are measured at the conclusion of part-time inpatient treatment (t1) and eight weeks post treatment (t2). The study will recruit participants diagnosed with depression from a day clinic at a hospital for mental health in Lower Saxony, Germany. The control group engage in the local in person eight-week aftercare groups offered by the psychiatric hospital. The participants in the intervention group engage in app-supported digital aftercare.

**Results:**

The results of the statistical data analysis are presented.

**Conclusions:**

The results provide insight into whether the app-based intervention achieves a comparable level of stabilization to local in-person eight-week aftercare groups. Feedback from the intervention on the use of the app is critically discussed with regard to possible obstacles.

**Disclosure of Interest:**

None Declared

